# Efficacy and safety of autologous platelet rich plasma for the treatment of vascular ulcers in primary care: Phase III study

**DOI:** 10.1186/s12875-014-0211-8

**Published:** 2014-12-30

**Authors:** Kepa M San Sebastian, Igone Lobato, Igone Hernández, Natalia Burgos-Alonso, Maria Cruz Gomez-Fernandez, Jose Luis López, Begoña Rodríguez, Anna Giné March, Gonzalo Grandes, Isabel Andia

**Affiliations:** Ezkerraldea-Enkarterri Health Region, Basque Health Service (Osakidetza), Portugalete, Spain; Primary Care Research Unit of Bizkaia, BioCruces Health Research Institute, Osakidetza, Luis Power 18, planta 4; E-48014, Bilbao, Spain; Family Medicine Teaching Unit, Bilbao, Spain; Bilbao Health Region, Basque Health Service (Osakidetza), Bilbao, Spain; Regenerative Medicine Laboratory, BioCruces Health Research Institute, Cruces University Hospital, Bilbao, Spain; Preventive Medicine and Public Health Department, University of the Basque Country, Faculty of Medicine and Odontology, UPV/EHU, Bilbao, Spain

**Keywords:** Vascular ulcer, Platelet rich plasma, Primary care

## Abstract

**Background:**

Vascular ulcers are commonly seen in daily practice at all levels of care and have great impact at personal, professional and social levels with a high cost in terms of human and material resources. Given that the application of autologous platelet rich plasma has been shown to decrease healing times in various different studies in the hospital setting, we considered that it would be interesting to assess the efficacy and feasibility of this treatment in primary care. The objectives of this study are to assess the potential efficacy and safety of autologous platelet rich plasma for the treatment of venous ulcers compared to the conventional treatment (moist wound care) in primary care patients with chronic venous insufficiency (C, clinical class, E, aetiology, A, anatomy and P, pathophysiology classification C6).

**Design:**

We will conduct a phase III, open*-*label*,* parallel*-*group, multicentre, randomized study. The subjects will be 150 patients aged between 40 and 100 years of age with an at least 2-month history of a vascular venous ulcer assigned to ten primary care centres. For the treatment with autologous platelet rich plasma, all the following tasks will be performed in the primary care setting: blood collection, centrifugation, separation of platelet rich plasma, activation of coagulation adding calcium chloride and application of the PRP topically after gelification. The control group will receive standard moist wound care. The outcome variables to be measured at baseline, and at weeks 5 and 9 later include: reduction in the ulcer area, Chronic Venous Insufficiency Quality of Life Questionnaire score, and percentage of patients who require wound care only once a week.

**Discussion:**

The results of this study will be useful to improve the protocol for using platelet rich plasma in chronic vascular ulcers and to favour wider use of this treatment in primary care.

**Trial registration:**

Current Controlled Trials NCT02213952

## Background

The term ulcer is used to refer to spontaneous or traumatic lesions, typically in the lower extremities, that do not heal in a reasonable time, with an underlying aetiology that may related to systemic disease or local disorders. The healing process is complex and dynamic [1]. Once ulcers start to be treated, they eventually heal completely in most cases. However, due to some risk factors associated with the patient (comorbidities) or to the ulcer (infection), acute ulcers can become chronic [2].

Vascular ulcers represent a common severe health problem. Several studies report a prevalence of 1 to 2% for venous ulcers in the general population [3-6], and this type of ulcer represents 75 to 80% of all vascular ulcers. From these data, it can be estimated that in Spain there are between 250,000 and 300,000 people with venous ulcers, implying that 300,000 to 375,000 people have vascular ulcers.

In line with these figures, vascular ulcers represent a common disorder in daily medical practice at all levels but especially in primary care (PC). They have a significant impact at the individual, professional and social levels with a high cost in terms of human and material resources. However, it is still not well understood why venous ulcers develop, research efforts being ongoing to establish new theories on their pathogenesis [7]. Further, the current approach to their care does not always achieve adequate healing of these ulcers, and hence a range of alternative treatments is being tested [8,9].

Autologous platelet-rich plasma (PRP) is a product derived from blood that is increasingly widely used in clinical practice and, among other applications, it has become an alternative to the dressings used to date for the treatment of vascular ulcers. The curative properties of PRP rely on the fact that platelets are a physiological reservoir of growth factors, which have an active role in tissue regeneration. It is well known that platelets contain a great variety of growth factors, with healing functions [10].

On the other hand, despite evidence on the effectiveness of autologous PRP in venous ulcers, the effectiveness and feasibility of this treatment in the general population in primary care remain unclear. Most of studies have been limited to small groups of the population, in private clinics and hospitals [11]. The Salazar-Alvarez et al. prospective study, which is done in PC on 11 patients, concludes that PRP is a valuable and practical procedure that promotes the healing of chronic ulcers of the lower extremity [12]. However, it is necessary to conduct a well designed controlled clinical trial, examining the effect ofPRP in both ulcer healing and the patients’ quality of life. For these reasons, we propose a phase III clinical trial.

## Methods and design

### Hypothesis

After 2 months, treatment with PRP will lead to at least a 20% greater reduction in the ulcer area than observed in patients receiving standard care based on the current protocol in PC of moist wound care (MWC).

### Objectives

#### Primary objectives

To assess the efficacy in healing the ulcers attributable to autologous PRP by estimating the change in the ulcer area using serial photographs, at baseline and 5 and 9 weeks after starting the treatment, compared with that in the control group.

### Secondary objectives

To assess the safety of autologous PRP for the treatment of venous ulcers in patients with chronic venous insufficiency corresponding to C6 on the CEAP classification (C, clinical class, E, aetiology, A, anatomy and P, pathophysiology of the disease), describing changes in patient perceived pain, the percentage of ulcers with infection, and related perilesional changes, in both groups and comparing the results.

• To estimate the quality of life of patients in terms of the Chronic Venous Insufficiency Quality of Life Questionnaire (CIVIQ) score in both groups at baseline, and 5 and 9 weeks after starting the treatment.

### Study design

We will conduct a phase III open*-*label*,* parallel*-*group*,* multicentre*,* randomized study. We will analyse 150 patients between 40 and 100 years old who have venous vascular ulcers and are assigned to ten health centres of the Basque Health Service (Osakidetza) in the Ezkerraldea-Enkarterri, Barakaldo and Bilbao health region. Two groups will be formed: one intervention group, to be treated with autologous PRP and a control group, to be treated following the current recommendations for MWC.

Treatment with autologous PRP involves five steps: blood collection, centrifugation, separation of PRP, activation of the coagulation process using calcium chloride and placement of the PRP dressing, covering it with a secondary dressing.

Given that it is not feasible to use a placebo that could blind those involved to the use of the autologous PRP, the control group will receive conventional treatment of MWC, which is the usual care provided in our health service, and this will be carried out in accordance with the recommendations of Basque Health Service (Osakidetza).

### Study setting

The study is to be conducted in the PC health centres of, eligible patients being all those with venous vascular ulcers aged between 40 and 100 years of age under the care of Ezkerraldea-Enkarterri, Barakaldo and Bilbao health region, these centres and currently being treated with MWC in accordance with the recommendations of Basque Health Service (Osakidetza).

### Inclusion criteria

To be included, patients should meet all of the following criteria:

- Resident in Barakaldo or Bilbao seen in the treatment rooms of the PC health centres of the Ezkerraldea-Enkarterri and Bilbao health region of Osakidetza, for MWC
- Aged between 40 and 100 years of age of either sex
- Chronic venous insufficiency corresponding to C6 on the CEAP classification
- Vascular ulcer that does not respond to conventional treatment in 2 months
- Normal blood test results in terms of platelet and red blood cell counts and haematocrit
- One or two ulcer together whose area is equal or less than 20cm^2^
- Patients with a negative analytical on syphilis, Hepatitis B, Hepatitis Cand HIV I/II
- Ankle Brachial Index ≥0.8 and ≤1.5
- Independent or with sufficient family support to travel to the health centre
- Informed consent form signed

### Exclusion criteria

Patients will be excluded if any of the following apply:

- Being pregnant or breastfeeding, or a woman of fertile age not using contraceptive methods
- Chronic use of immunosuppressants or antiretroviral drugs
- Patients with syphilis, Hepatitis B, Hepatitis Cand HIV
- Clotting disorders
- Chronic infectious diseases
- Treatment with radiotherapy or chemotherapy
- History of cancer
- Active infection or fever at the beginning of the study
- ABI below 0.8 or above 1.5
- People who are taking a drug under clinical investigation or participated in any study under clinical investigation in the previous 30 days

### Reasons for withdrawal from the trial

Patients are free to withdraw from the trial at any time. If patients develop signs of infection with a visible exudate, they will be assessed by the research team, and it will be decided, together with the patient whether or not they should continue in the study, this event being recorded in the final report. In addition, they will be withdrawn if any of the following apply:

- Poor course of the ulcer with significant oedema, signs of regional infection, lymphangitis, cellulitis or excessive pain
- Wound care management by people other than those on the research team
- Inability or failure to attend scheduled appointments
- Problems with vascular access
- Inability to travel to the health centre by themselves or insufficient family support (coming to light after the start of the trial)
- Meeting any exclusion criteria during the course of the study
- Any other problems that in the opinion of the research team justify treatment withdrawal.

### Recruitment

Patients will be recruited by the representatives of the ulcer commission of the health region or, in the absence of such a figure, the person appointed for this in each of the participating centres. Before starting the study, all those involved will be contacted to explain the study methodology.

**Figure 1:**
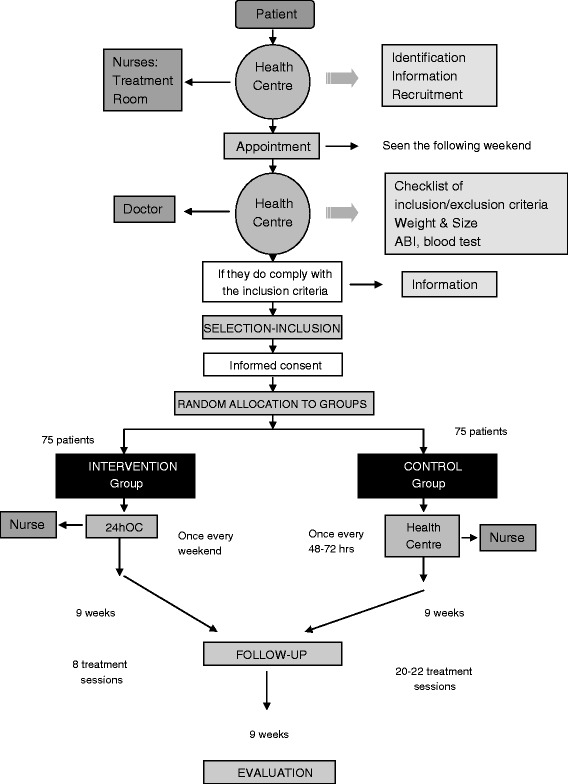
**Study algorithm.**

The recruitment process is to start in the treatment rooms of the health centres (Figure 1). Each of aforementioned individuals responsible for this process will have access to the necessary information for consecutively recruiting patients in line with the inclusion and exclusion criteria. When eligible patients are identified, the nurses will explain the possibility of their inclusion in the study and if they agree to participate, they will be referred to the assigned outpatient clinic, a corresponding appointment being arranged for them.

Patients will have an appointment in the health center; they will be seen by a doctor of the centre, who will carry out an interview to collect data related to the selection check list, and they will be weighed, their height measured, and a ABI test will be performed. If the patient meets the inclusion criteria, they or their legal guardian will be asked to give verbal and written informed consent before inclusion in the clinical trial, and after a “Patient Information Sheet for research projects involving the use of biological samples” has been provided and explained to them, by the principal investigator.

Once patients give informed consent, the doctor will contact the Primary Care Research Unit of Bizkaia to request that the patient be randomly allocated to the control or intervention group, and their inclusion in the study will be reported to their general practitioner. The patient’s wound will be cared for as usual, and they will be given a patient handbook including a diary to keep a daily record of their experiences and any adverse effects, and an appointment will be made for their first treatment as part of the study, either using autologous PRP or under the MWC protocol, depending on their group allocation. The patient handbook also includes a telephone number they should call in the event of any unforeseen problems between treatments.

In the event that there is no record in a patient’s medical record of a blood test having been performed in the 6-months prior to inclusion in the study, the general practitioner will request a blood test, the results of will be taken as the baseline measurement.

### Protocol

#### Control group

Patients in the control group will be treated as they had been prior to inclusion, that is, two to three times a week and in accordance with the health region recommendations, namely to maintain a moist healing environment. The wound care will consistently be provided by the same nurse, who will be responsible for making the appointments within the available timetable in the treatment room. The data will be registered in an Access database by the administrator. The treatment schedule for the control group is displayed in Tables 1 and 2.

**Table 1:** **Timetable for the control group**

**Activities**	**Recruitment Day 0**	**Selection Day 1**	**Day 3**	**Day 5**	**Day 8**	**Day 15**	**Day 22**	**Day 29**	**Day 36**	**Day 43**	**Day 50**	**Day 57**	**Day 64**
Patient recruitment	X												
1^st^ visit		X											
Leg Doppler ultrasound		X											
Informed consent		X											
Randomisation		X											
Inform GP in writing		X											
Conventional treatment		X	X	X									
Autologous PRP treatment					X	X	X	X	X	X	X	X	
Ulcer Assessment					X				X				X
Final Assessment													X

**Table 2:** **Timetable for the intervention group**

**Activities**	**Recruitment Day 0**	**Selection Day 1**	**Day 3**	**Day 5**	**Day 8**	**Day 15**	**Day 22**	**Day 29**	**Day 36**	**Day 43**	**Day 50**	**Day 57**	**Day 64**
Patient recruitment	X												
1^st^ visit		X											
Leg Doppler ultrasound		X											
Informed consent		X											
Randomisation		X											
Inform GP in writing		X											
Conventional treatment		X	X	X									
Autologous PRP treatment					X	X	X	X	X	X	X	X	
Ulcer Assessment					X				X				X
Final Assessment													X

Week^(1)^: the wound will be treated every 48 to 72 hours during these weeks, as required in each case (depending on the condition of the ulcer).

Week^(1)^: the wound will be treated every 48 to 72 hours during these weeks, as required in each case (depending on the condition of the ulcer).

### Moist wound care

Patients in the control group will be treated following the recommendations for MWC of Osakidetza (described in the rational use of the moist wound care products section of the Osakidetza 2011 continuous professional education guide). Material is selected for wound care depending on the assessment of the bed of the wound and surrounding skin, as well as the type and amount of exudate and whether there are signs of infection. These wound care treatments will be carried out every 48–72 hours, in line with the current usual practice.

There are multiple types of dressings, each of which have a proposed indication as well as certain known advantages and disadvantages. It is important to recognise that no single dressing is suitable for all types of ulcers and few are indicated for all stages of healing. For a given wound, the ideal dressing should absorb excess exudate, create a moist environment, and maintain an adequate pH, while also being thermally insulating, impermeable to bacteria and easy to change, as well as not causing allergic reactions or damage when removed.

The materials used in the MWC include the following:

- Debriding agents: enzymatic, mechanical, physiological and autolytic
- Antimicrobial agents: containing silver, indicated for infected wounds
- Activated charcoal: for odour control
- Hydrocolloids: for debridement and promotion of granulation tissue, one of their strengths being that they are not very absorbent.
- Alginates and hydrofibres: for exudating and contaminated wounds and they also have a hemostatic effect; a disadvantage of alginates being that they are less absorbent than hydrofibres
- Polyurethane foams: hydrocellular dressings and hydropolymer dressings; the latter are indicated for moderate to high amounts of exudate, but their disadvantages include that they do not absorb exudates or debride the wound in the same way.
- Barrier products: for preventing maceration and irritation of the skin around the lesion

When selecting a dressing, as well as the characteristics of the wound, it is necessary toassess the general status of the patient, the availability of resources, and the cost-effectiveness of alternatives, as well as how easy it is to apply.

### Intervention group

Patients in the intervention group will receive wound care once a week with autologous PRP, based on the estimated mean lifespan of platelets (7–10 days). The treatment with autologous PRP will be performed in the assigned outpatient clinic, in the phlebotomy room, by qualified nurses (IL, IH, BR).

As in the control group, the nurse will be responsible for making appointments for the next treatment and will collect data to be entered in an Access database by the administrator. The first treatment session of the study will be performed on day 8, and the data will be recorded in the patient medical record. From this point onwards, data will be collected every 7 days for 9 weeks. Treatment schedule for the PRP group is shown in Table 2.

### Outcome measures

#### Reduction in ulcer size

A main variable, in terms of the difference in the surface area of the ulcer at weeks 5 and 9 of treatment compared to baseline, expressed as the percent reduction in area for each of the patients in the intervention and control groups.

#### Ulcer area

Surface area of the ulcer, expressed in square centimetres and measured at baseline and at weeks 5 and 9, using the ImageJ software to analyse photographs taken at these time points.

#### CIVIQ score

Score on this specific scale for assessing the quality of life of patients with chronic venous insufficiency, considering four dimensions (physical, psychological, social and pain). This secondary variable will be measured using a questionnaire for the patient, yielding a score between 20 corresponding to the lowest quality of life, and 100, the best quality of life. The questionnaire will be administered and the score recorded by the nurse in the baseline treatment session, and again at weeks 5 and 9.

#### Safety

Will be assessed in terms of any adverse effects recorded by patients and by the weekly interview carried out by the nurse, who will ask about the pain, assess the degree of infection and the type of exudates, as well as any adverse effects associated with the treatment, comparing with the results in the control group.

### Adverse effects

At all of the appointments during the study period, the nurse will ask the patients whether they have experienced any adverse effects. Any effects identified will be recorded by the nurse himself/herself in the database created for the purpose, with the following data: severity and potential relationship with the study treatment. In addition, there will also be a committee independent of the research team that will monitor the safety of the treatment under study and review any adverse events that do arise during the study.

Complying with the current legislation, any serious adverse reaction (SAR), or unexpected serious adverse reaction, (USAR) will be reported to the health authorities within 24 hours of being detected.

### Follow-up period

The follow-up period will last for 9 weeks, but all the adverse effects that appear within 30 days of stopping the treatment will be recorded.

### Sample size

The estimated sample size is 150 patients, based on a previous meta-analysis by Martinez-Zapata et al. [13]. The patients will be randomly allocated to the intervention (treatment with PRP) or the control group (standard moist wound care), in a 1:1 ratio which involves 75 patient per group. The calculation is based on a clinically meaningful difference of at least 15 points between groups, for α = 0,05 and β = 20%, and considering 30% of withdrawal.

### Statistical analysis

The categorical variables will be presented as frequencies and percentages (for example, the percentage of patients requiring ≤1 weekly treatment) and continuous variables using measures of central tendency and dispersion (mean and standard deviation for normally distributed variables, and otherwise median and interquartile rank).

The analysis of the efficacy will be carried out on an intention-to-treat basis by comparing the percent reduction in ulcer area, observed in each of the groups after 5 and 9 weeks of treatment, with respect to baseline. Using the Mann–Whitney U non-parametric test, we will estimate the effect attributable to the intervention using the difference between the changes occurring in the two groups.

Subsequently, in order to adjust the comparisons for the baseline measurements, we will carry out covariance analysis models. As well as adjusting for baseline, we will adjust for the potential confounding or effect-modifying covariates. A p value <0.05 will be considered statistically significant.

All the analyses will be performed using SAS version 9.2 statistical software.

### Quality control and assurance

Quality control will consist of collecting data on adherence to the intervention, patient inclusion and follow-up, as well as the monitoring the quality of data entry.

Monitoring: This study will be monitored Veronica Arce, a nurse who works for the Primary Care Research Unit of Bizkaia, a body that provides methodological and statistical support for the trial; she will perform two checks per patient, in the first and last visit.

If there were to be suspicion of a serious violation of the trial protocol, which would imply a significant effect on:

- the physical or psychological integrity or safety of patients during the trial, or
- the values of the study,

the study sponsor will be contacted as soon as possible. In any case, all violations will be notified to the relevant authorities in accordance with current legislation.

### Legal and ethical issues

This research project will be carried out in accordance with the principles of the Declaration of Helsinki and in compliance with current legislation on medical research.

Prior to carrying out the study, approval will be sought from the Management of the Ezkerraldea-Enkarterri Health Region as well as consent from the participating patients, guaranteeing that the anonymity and confidentiality of the data collected will be safeguarded as provided for in the Spanish Organic Act 15/1999, of December 13, on Personal Data Protection. Any substantial changes to the original documents will be submitted to the Ethics Committee and/or the regulatory authority for approval.

The trial staff will ensure the preservation of the anonymity of patients. Patient records will be coded with 5 digits: the first referring to the outpatient or health centre, the second to the ulcer and the last three to their order of inclusion.

### Limitations of the study

Taking into account that the treatment applied to both groups consists initially of a topical treatment, with significant differences in the appearance of the dressing to be applied, patients will be aware of their group allocation, as will the researchers, meaning that it is not feasible to double blind the study. To prevent the results of the research being influenced by observer bias, the progress of the ulcers will be assessed by someone independent of the research team, who will have exclusive access to photographs of the ulcers, with no type of identifying data related to the patient or their group.

## Discussion

This study has two main objectives efficacy and safety of autologous PRP, while we also hope to explore its effect of quality of life and cost-effectiveness. Prior to determining the efficacy of PRP, we consider it important to investigate multiple factors related to the feasibility of using this treatment in the PC setting. We believe that the autologous PRP-based approach may be able to reduce the costs associated with the management of this type of health problem. Venous ulcers treated by conventional wound care (in accordance with the recommendations of the MWC of Osakidetza) take at least 10–12 weeks to heal, meaning significant financial and personal costs as well as impairment in the quality of life of patients with daily treatments and risk of infection associated with the daily management of ulcers. Preliminar studies show that PRP is a safe, simple and effective procedure in treating chronic venous ulcers [14]. We presume that using PRP treatment it would only be necessary to clean the wound repeat the PRP application procedure once a week, since the mean lifespan of platelets is 7–10 days. With this study, we will be able to assess whether this technique could be cost-effective in PC.

Autologous PRP treatment is used for managing various types of lesions including corneal, vasculitic, neuropathic and diabetic foot ulcers, among [15,16] others. Several studies using this technique have demonstrated a decrease in healing time compared to conventional treatments (in particular, the currently recommended MWC), though no studies have been undertaken in PC [7]. However, a recently published meta-analysis [16] has underlined the variability between the results published to date, concluding that it is not yet possible to recommend this type of treatment due to the lack of rigorous studies with representative sample sizes. Further, the Spanish Agency of Medicines and Medical Devices that regulates the use of PRP has encouraged researchers to continue investigating this treatment with high quality protocols and representative samples to help to ascertain whether it is effective [17].

The high proportion of the population seen at the first level of care (i.e., PC) means that this setting provides great opportunities for carrying out social and health research and establishing preventative measures that can be extrapolated to the entire community: in any intervention measure or research project, the closer to day-to-day reality, the better the application in clinical practice [14]. This is particularly true in the case of ulcers, the majority of this type of lesion being cared for in PC.

## Abbreviations

PRP: Platelet rich plasma
CEAP: Classification: C, clinical class, E, aetiology, A, anatomy and P, pathophysiology
CIVIQ: Chronic Venous Insufficiency Quality of Life Questionnaire
PC: Primary care
MWC: Moist wound care
